# Anal Cancer Screening and High‐Resolution Anoscopy: What Surgeons Should Know

**DOI:** 10.1111/ans.70873

**Published:** 2026-07-20

**Authors:** Matthew Joseph Marino, Benedict Blacket, Richard Clive Turner, Tamzin Cuming, David R. C. James

**Affiliations:** ^1^ Oxford University Hospitals Trust Oxford UK; ^2^ Austin Hospital Heidelberg Victoria Australia; ^3^ Epworth Hospital Melbourne Victoria Australia; ^4^ University of Tasmania School of Medicine Hobart Tasmania Australia; ^5^ Royal Hobart Hospital Hobart Tasmania Australia; ^6^ Homerton Anal Neoplasia Service, Homerton Health Care NHS Trust London UK

## 1 Introduction

Anal squamous cell carcinoma (ASCC) is considered a rare cancer; however, its incidence is increasing. In certain patient populations such as HIV‐positive men who have sex with men (MSMHIV), the incidence is high, at 85/100 000 [1]. This is higher than the general population incidence of colorectal cancer at 75/100 000 [2]. Population screening for colorectal cancer is common, yet anal cancer screening in these high‐risk populations is not widespread. Two‐thirds of ASCC occur in immunocompetent women, with an incidence of approximately 2/100 000 [2, 3]. Almost all ASCC is associated with high‐risk human papillomavirus (HR‐HPV) infection, up to 98% of cases in MSMHIV, particularly subtype 16 [4].

Despite widespread HPV vaccination and access to antiretroviral therapy, ASCC incidence and mortality are rising globally, particularly in the UK and Eastern Europe, with most disease occurring in developed countries [3, 5, 6]. According to the National Cancer Institute's Surveillance, Epidemiology, and End Results (SEER) database, age‐adjusted rates for new anal cancer cases have risen on average by 2.2% per year from 2013 to 2022. Death rates have increased on average by 4.1% per year from 2014 to 2023 [7].

HPV vaccination is expected to significantly impact ASCC burden; however, it is unlikely to be observed for decades due to lag time between HPV infection and the development of ASCC (approximately 10–20 years), and suboptimal global HPV vaccination coverage [3]. This is especially true in males, who in 2020 were included in only one‐third of the world's HPV vaccination programmes, often commencing later than those for females [8]. Recent evidence supporting the vaccine's efficacy comes from a Danish nationwide cohort study of women aged 17–32 years. Among 968 881 women, 37 cases of anal high‐grade squamous intraepithelial lesions (HSIL) or worse occurred among the unvaccinated, 26 in women vaccinated between 17 and 32 years, and fewer than five in women vaccinated before 17 [9].

HSIL, also known as high‐grade anal intraepithelial neoplasia (AIN), is a precursor to ASCC and can be detected using high‐resolution anoscopy (HRA) with biopsy [10, 11, 12]. These lesions are usually asymptomatic unless advanced, raising concern for early invasion. Just as pre‐cancerous cervical lesions can be screened and treated, the same applies to anal HSIL. Historically, AIN was treated by ‘anal mapping’ in which patients were periodically examined under anaesthetic and biopsied systematically around the anus to exclude early invasion. This practice was non‐specific and left patients with scarring of the perianus with minimal benefit. Although there is a paucity of evidence directly comparing standard anoscopy to HRA, the latter enables targeted biopsy and has long been practised in some centres [13]. Interest has grown following the 2022 Anal Cancer/HSIL Outcomes Research (ANCHOR) randomised controlled trial, which found a 57% reduction in cancer progression among people living with HIV (PLWHIV) treated for HSIL [14].

Subsequently, the International Anal Neoplasia Society (IANS) developed consensus screening guidelines in 2024, recommending screening for high‐risk groups [15, 16]. Whilst HRA can be used as a screening tool alone (as in the ANCHOR study), initial screening includes digital anorectal examination (DARE) with anal cytology and/or HR‐HPV testing. Those with abnormal results are then referred for diagnostic HRA and, if HSIL is confirmed histologically, for HRA‐guided treatment. HR‐HPV testing alone has the highest HRA referral rate—up to 75% in MSMHIV [17] but is likely lower in women [18]. Cytology screening can be used to triage those with HR‐HPV, maintaining sensitivity whilst improving specificity and reducing referral rates by around 20% [19]. DARE alone can serve as a screening test for ASCC when HRA is unavailable because most HSILs are impalpable.

Screening guidelines have been developed in Australia for PLWHIV [15], and it is expected that local guidelines will continue to be rolled out in other countries. A limited number of trained HRA providers restricts access to screening for high‐risk patients. With referral to HRA rates in men who have sex with men (MSM) ranging between 20% and 70% [17], the number of patients requiring HRA in this group alone is likely to surpass capacity before other at‐risk cohorts are considered. Therefore, in addition to improved screening biomarkers to reduce referrals for HRA, there is a pressing need to train clinicians in HRA.

While HRA is practised across various specialties, it has not been widely adopted by surgeons. Colorectal surgeons are well‐positioned to play a leading role in the provision of HRA due to familiarity with anorectal anatomy, technical skills (especially when suspicious lesions require excision), and access to the operating theatre when needed. Surgeons typically oversee anal cancer management and lead colorectal MDTs, where these patients are discussed. Involvement in the provision of HRA and anal cancer screening complements the comprehensive care offered from diagnosis to surveillance and salvage.

Rather than a systematic review, this article presents a narrative review informed by available clinical trials, observational studies, international guidelines, and expert consensus. While recent developments, particularly the ANCHOR trial, have provided important evidence supporting treatment of HSIL in PLWHIV, the evidence base for screening and treatment in other populations remains evolving. Accordingly, the recommendations discussed reflect current best practice and expert interpretation of available data, while acknowledging important uncertainties in screening strategies, long‐term outcomes, and cost‐effectiveness in non‐HIV populations.

## 2 High‐Resolution Anoscopy—The Procedure

HRA is defined as ‘the examination of the anal canal and perianus using a colposcope for lighting and magnification, after application of 5% acetic acid and Lugol iodine solutions to identify anal lesions’ [20]. The procedure begins with patient counselling and informed consent, before the patient is positioned in left lateral or lithotomy.

A cytobrush or dacron swab, moistened with tap water, is then used for anal cytology and HPV testing. To enhance cytology quality this is done before DARE and without lubrication. Advising patients to refrain from rectal cleansing procedures (‘douching’) or receptive anal intercourse for 24 h prior also improves cytology yield.

A cotton‐tipped swab is used to liberally apply 5% acetic acid to the anal canal and perianus. This is left for 3–5 min to allow maximal demarcation. Acetic acid demarcates the ‘squamocolumnar junction’ (SCJ), with squamous cells of the anal canal becoming slightly white and rectal columnar mucosa remaining the same. The colposcope is focused, and magnification is increased to systematically examine the anal canal from just proximal to the SCJ to the anal verge. Acetic acid‐soaked cotton‐tips are used to open folds and ensure the entire surface is examined. The perianus is then systematically assessed. This process usually takes between 5 and 15 min for a trained practitioner [20].

### 2.1 Diagnostic HRA

A diagnostic HRA is performed on initial consultation or during surveillance. Suspected HSIL is biopsied with suitable forceps after infiltration of local anaesthetic (LA) when required. Photographs are taken and findings documented, clearly outlining the appearance (uptake of acetic acid, response to Lugol's iodine, margins, contour, vascular pattern and epithelial changes), size and location of the biopsied lesions. Diagrammatic templates for both the anal canal and perianus are available and should be used for consistency and reproducibility.

**FIGURE 1:**
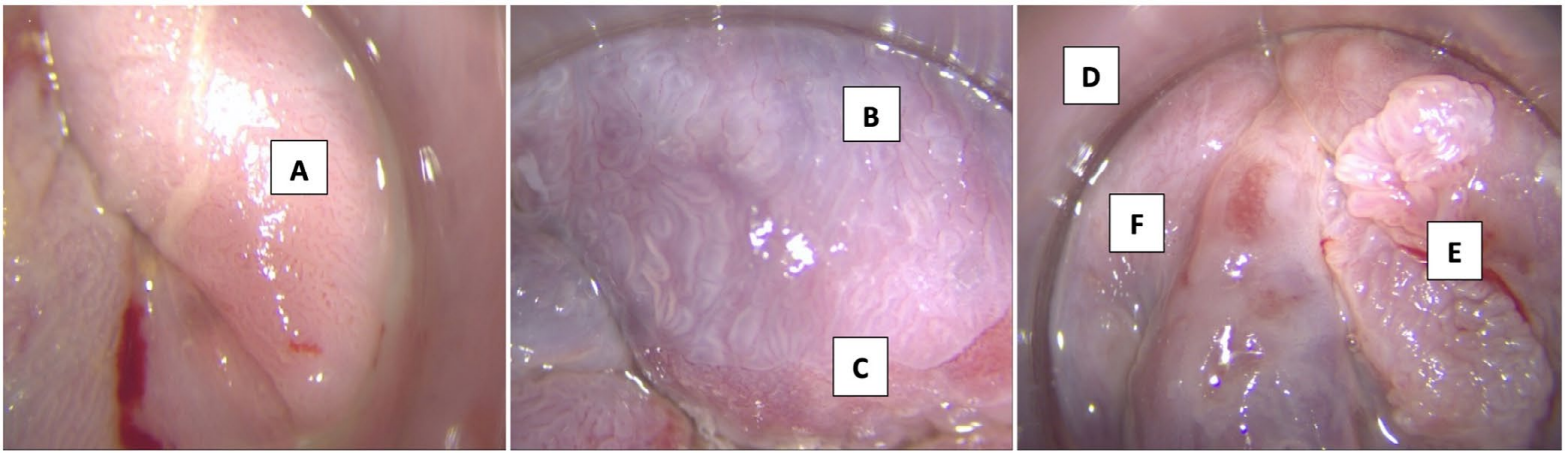
Appearance of low‐ and high‐grade squamous intraepithelial lesions taken at high‐resolution anoscopy. (A) Common appearance of HSIL. This lesion is strongly acetowhite, flat with indistinct margins, coarse punctations and striations. (B) Classic demonstration of mosaicism, a common finding in HSIL. (C) Demonstration of the squamocolumnar junction (SCJ). Pink‐coloured tissue is rectal columnar mucosa. Squamous cells of the anal canal take up acetic acid and appear waxy‐white. (D) Edge of the anoscope. (E) LSIL—classic appearance of anal condyloma acuminatum, consisting of an acetowhite, raised lesion with loopy vessels. (F) Subtle area of HSIL. It is not uncommon to find HSIL and LSIL simultaneously.

Acetic acid is strongly absorbed by dysplastic cells that have high nuclear density, producing ‘acetowhite change’, while these immature, glycogen‐poor cells do not take up Lugol's iodine. Acetowhite staining also accentuates abnormal vascular patterns caused by vessel distortion from rapidly proliferating cells. These patterns are recognised as coarse red dots (punctation) and lines (striations) (Figure 1). Using the green filter on the colposcope may assist with identification of vascular anomalies.

Fine punctation and striations are characteristic of low‐grade squamous intraepithelial lesions (LSIL) (Figure 1), which are often flat. HSIL strongly takes up acetic acid, turning intensely acetowhite and appearing pale yellow with the application of Lugol's iodine (vs. dark brown for normal or LSIL), and may demonstrate features such as mosaic change (erratic paving patterns) (Figure 1).

Educating surgeons to recognise these patterns is a central component of HRA training [21]. Anoscopists, especially those new to the practice, should maintain a low threshold to biopsy any tissue suspected of harbouring HSIL. Emerging artificial intelligence tools for HSIL detection are under development and will likely augment HRA practice [22]. Anoscopists play a role in contributing to the development of neural networks.

### 2.2 Treatment HRA

HSIL treatment can broadly be divided into topical or ablative therapy. Targeted, HRA‐guided ablation is performed using electrocoagulation (most familiar to surgeons), CO_2_ or diode laser, or radio‐frequency ablation (RFA) [23]. Trichloroacetic acid is a topical ablative agent that is applied by the clinician under HRA guidance. Other topical therapies, such as imiquimod and 5‐fluorouracil, can be self‐applied by the patient to treat HSIL or downsize widespread disease before HRA‐guided ablation.

A treatment HRA involves HRA‐guided ablation of histologically confirmed HSIL. Previous photographs and documentation help guide the anoscopist to confirmed disease, which is treated accordingly, usually after instillation of LA.

After a period of supervised recovery, the patient is discharged with post‐operative advice, and a follow‐up appointment is made with timing dependent on whether a diagnostic or treatment HRA was performed.

Simple analgesia usually suffices during recovery if only small areas have been treated. If more than one quadrant has been treated, laxatives and topical analgesia (gel or ointment containing lignocaine) are additionally considered. Treatment of widespread disease is often staged to limit pain and stenosis risk.

Because a proportion of screen‐detected HSIL can spontaneously regress over 6–12 months [24], topical therapy or a watch‐and‐wait approach with short‐term follow‐up could be considered in low‐risk patients. This is not an option in high‐risk patients and those with a high burden of disease (over 50% of the anal canal or perianus). A dedicated HSIL multidisciplinary team meeting helps manage complex patients.

### 2.3 Setting

HRA is often performed in the outpatient setting under LA only. A dedicated clinic space or office large enough to accommodate all necessary equipment and personnel is essential, and when well configured, facilitates an efficient flow of patients with high turnover. An ensuite bathroom helps maintain dignity for patients in whom the procedure creates an urge to open their bowels. Bowel preparation is not required and can prevent accurate cytology.

Some patients require treatment in the operating theatre. These include patients who will not tolerate the procedure under LA (such as victims of sexual assault or anal stenosis from prior radiation), patients requiring extensive treatment, multi‐zonal disease (concurrent squamous intraepithelial lesions affecting the genitalia) or concurrent procedures. See Table 1 for the advantages and disadvantages of HRA under LA or GA. The area requiring treatment can be spot‐marked in a prior HRA session by injecting a small amount of tattoo submucosally beneath the lesion, although this can be technically challenging.

**TABLE 1:** Indications, advantages and disadvantages of high‐resolution anoscopy under local versus general anaesthesia.

	Local anaesthesia (LA)	General anaesthesia (GA)
Indications	Short procedure (e.g., surveillance HRA) Patients who have previously tolerated HRA under LA Better for diagnostic HRA High‐risk GA	Prolonged procedures (e.g., initial HRA where multiple biopsies likely required) Multi‐zonal disease Extensive treatment required Patients with previous sexual trauma who would not tolerate procedures awake Patients with anal stenosis (e.g., previous radiotherapy/aSCC surveillance)
Advantages	Quicker recovery No hospital admission required Suitable for clinic/outpatient setting Higher throughput and patient turn‐around Avoids GA risk/perioperative complications Cheaper	Better tolerated, especially in patients with previous bad experience and promotes compliance with surveillance HRA's Quicker procedure time when multiple biopsies/extensive ablation required Easier for new anoscopists and promotes better visualisation
Disadvantages	Greater patient discomfort/pain Patient movement Negative experience under LA may reduce likelihood of subsequent HRA	Requires anaesthetist and theatre staff GA/airway risks for arguably a quick and minor procedure Higher cost to healthcare system Higher cost to patient may reduce likelihood of subsequent HRA

### 2.4 Equipment

**FIGURE 2:**
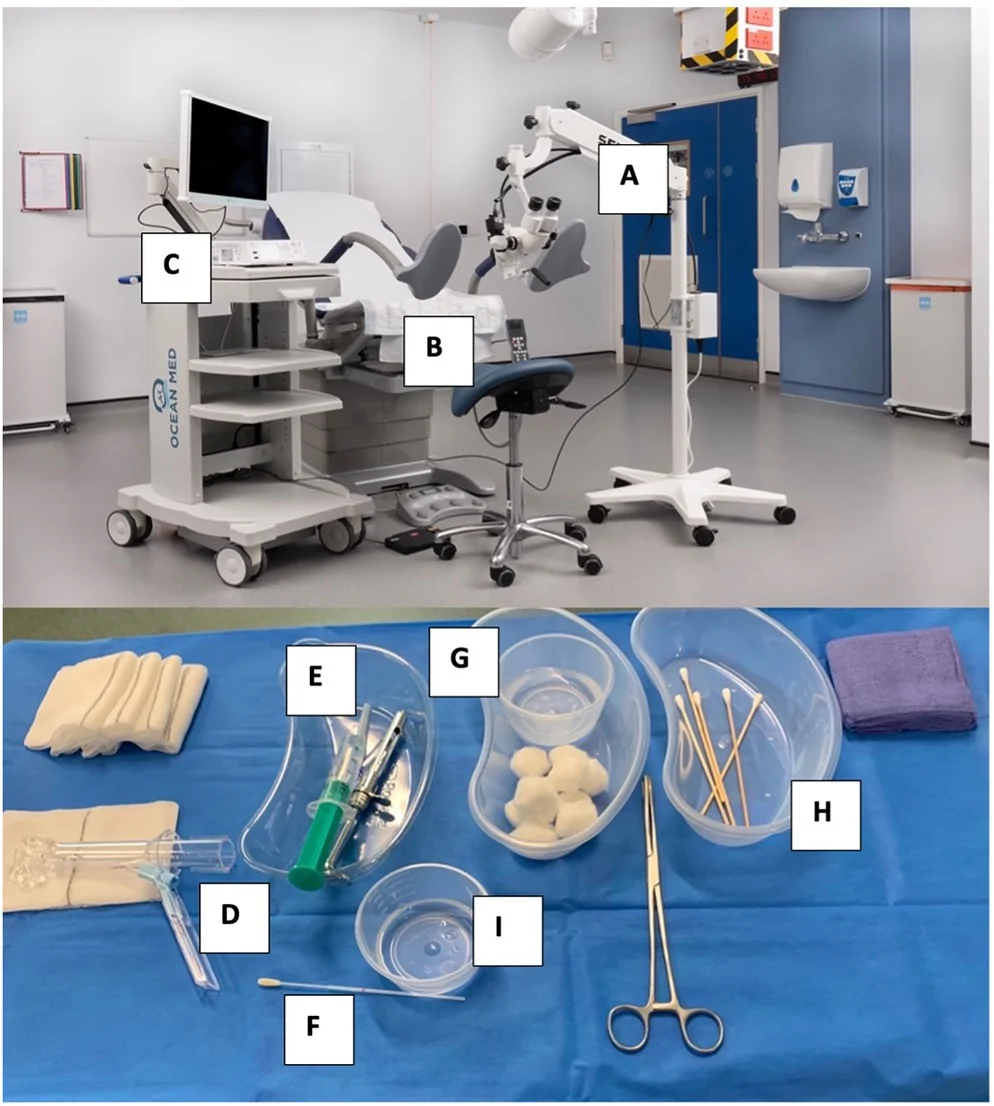
Equipment used for high‐resolution anoscopy. (A) Colposcope. (B) Lithotomy bed. A flat bed can also be used if performing anoscopy in the left‐lateral position. (C) Video monitor and recording equipment. The anoscopist has the option to view the images through the colposcope eyepiece or on the monitor. (D) Anoscope. (E) Dental needle and syringe used for LA infiltration. (F) Cytology (Floq) swab. (G) Acetic acid. (H) Small cotton‐tipped swab. (I) Saline, used to moisten the cytology swab before insertion.

Equipment for HRA can be divided into fixed and single‐use. Fixed equipment (Figure 2) comprises reusable items that account for the majority of expenditure when establishing a service. The institution may already have some of these items being used by other services.

#### 2.4.1 Fixed Equipment

- Bed: Any flat examination bed will suffice for HRA performed in the left‐lateral position. A specific chair with stirrups is required for HRA in lithotomy.
- Microscope: A standard colposcope used by gynaecologists is sufficient to perform HRA. It must have sufficient magnification and adequate lighting.
  – Digital anoscopy is an emerging technique that uses an all‐in‐one, often disposable anoscope with an integrated camera to capture high‐resolution images using digital, rather than optical, magnification. As a colposcope is not used, the image is viewed solely on a screen instead of through eyepieces. Both techniques utilise the same steps and require acetic acid/Lugol's iodine. Digital systems may facilitate quicker adoption by colorectal surgeons experienced in endoscopy and improve audit and training, but they carry higher equipment costs and have limited outcome data compared with the established literature, which uses standard colposcopy [25].
- Air extractor: Extraction equipment is required to minimise potential transmission of aerosolised HPV generated during ablative therapy.
- Treatment equipment: A variety of ablative therapies are available, and the choice is anoscopist‐dependent, with no evidence of superiority between modalities [26]. Electrocoagulation and CO_2_ laser are the most common ablative methods, and both can be used in the outpatient setting. Laser is more precise but requires training and additional safety equipment.
- Video and photography equipment: Most modern colposcopes have video attachments that can be wired to a monitor, allowing visualisation on both the screen and eyepiece (standard colposcopy). Photographs taken during diagnostic HRA enable disease mapping.

#### 2.4.2 Single‐Use Equipment

- Swabs for anal cytology (Floq swabs) and pots for liquid‐based cytology (ThinPrep).
- Small histology jars and formalin.
- Anoscope: these are most commonly single‐use plastic (Figure 2) but can be autoclavable metallic instruments (cannot be used with a laser—unless the surface is deglossed). The handle of an anoscope is used to torque the scope and open folds, facilitating 360‐degree visualisation of the squamocolumnar junction (SCJ).
- Lubricant.
- 5% acetic acid: allowing identification of the SCJ and HSIL.
- Cotton‐tipped swabs: used to open mucosal folds and apply acetic acid and Lugol's iodine.
- Lugol's (aqueous) iodine: used to help assess equivocal lesions.
- Local anaesthetic and analgesia: As a minimum, lignocaine‐infused lubricant should be used liberally throughout the procedure. Targeted LA should be used submucosally for distal anal or perianal lesions before biopsy or treatment. Lesions proximal to the dentate line are likely insensate; however, this must be checked. Long, narrow dental needles and syringes are used as they fit down the anoscope without obscuring the view. Pre‐medication can be considered in those requiring extensive treatment or who tolerate HRA poorly: oral analgesia and/or topical anaesthesia (such as lignocaine/prilocaine—‘Emla’ cream) can be taken or applied before the procedure.
- Biopsy equipment: Baby Tischler forceps are commonly used to biopsy lesions within the anal canal, as they are long, narrow and easily pass through the anoscope to take superficial bites. They can be disposable or reusable. Perianal lesions can be biopsied with these or 2 mm punches, and small exophytic lesions can be excised for histology.
- Haemostatic equipment: Bleeding is uncommon; however, deep biopsies, especially when overlying haemorrhoids, can bleed significantly. Pressure is usually all that is required, but Monsel's solution (ferric subsulfate) can be applied. Although uncommon, Monsel's may stain the skin and is generally avoided on the perianus, where silver nitrate can be used instead. Diathermy and absorbable haemostatic products (such as Spongostan) can be used for significant bleeding.

### 2.5 Personnel

A nurse is required to assist with HRA, acting as both a scrub nurse and chaperone. Occasionally, other specialists may perform concurrent assessment in multi‐zonal disease, and anaesthetic staff are required when performing HRA under general anaesthesia in the operating theatre.

Ideally, specimens should be analysed by specialist histopathologists with experience in assessing anal or gynaecologic squamous intraepithelial lesions (SIL). The Lower Anogenital Squamous Terminology (LAST) provides a standardised nomenclature for HPV‐related lesions [27].

### 2.6 Scheduling

In general, HRA appointments take approximately 40 min, although new anoscopists will naturally require more time. Established anoscopists generally see four to five patients per session, depending on the mix of diagnostic or treatment HRAs and multi‐zonal assessments. New patients and treatment sessions take longer. Performing only diagnostic or treatment HRAs in the same session can improve efficiency.

### 2.7 Screening and Referral‐Base

Referrals for HRA arise from three pathways: as part of anal cancer screening, when HSIL is found incidentally, and as part of surveillance. Referrals can be expected from General Practitioners, Sexual Health and HIV specialists, Transplant Physicians, Gynaecologists, and General/Colorectal Surgeons.

IANS have developed consensus guidelines to inform anal cancer screening amongst various high‐risk groups. These include PLWHIV, MSM and transgender women (TW), patients with prior vulval dysplasia or cancer, and solid organ transplant recipients (Table 2). Others at risk, including the immunosuppressed, those with a history of cervical/vaginal HSIL or cancer, and anal condyloma, should be considered for screening with shared decision‐making, depending on access to HRA [16]. Whilst access to trained HRA providers is limited, only those in the highest risk groups (considered a 10‐fold greater incidence compared with the general population) should be screened.

**TABLE 2:** International Anal Neoplasia Society populations for screening—risk category A in whom screening is recommended.

HIV status	Population—risk category	When
With HIV	MSM and TW	Age 35
	Women	Age 45
	MSW	Age 45
Without HIV	MSM and TW	Age 45
	History of vulval HSIL or cancer	Within 1 year of diagnosis
	SOTR	10 years post‐transplant

*Note:* IANS guidelines on screening patients at the highest risk group for developing anal cancer [16].

Abbreviations: HSIL, high‐grade squamous intraepithelial lesions; MSM, men who have sex with men; MSW, men who have sex with women; SOTR, solid organ transplant recipients; TW, transgender women.

Patients with HSIL diagnosed incidentally (e.g., on biopsies taken at retroflexion during colonoscopy or on histology of haemorrhoids or skin tags) should undergo an HRA to look for residual disease and be managed accordingly. Surveillance following HSIL diagnosis is based on age, risk factors and the results of previous HRAs.

## 3 Training and Quality Metrics

Whilst the anatomy and certain technical components of the procedure may be familiar to many surgeons, HRA is unlike other procedures performed by General and Colorectal Surgeons. The practice is most similar to cervical colposcopy, but poses additional challenges, including uneven topography, haemorrhoids, folds and stool [28]. Unique to HRA within the context of colorectal surgery is the visual pattern recognition required to identify HSIL and differentiate lesions requiring treatment from LSIL and normal tissue.

Training comprises a theoretical and practical component. Courses run by various organisations and industry give a grounding in the theoretical element of HRA. There are multiple available, primarily online. IANS offers an online beginner's (standard) course, which is comprehensive, relevant and appropriate for surgeons, as well as an advanced course for those with HRA experience. One‐ or two‐day hands‐on courses are available in various countries, including Spain, the UK and the US. In 2026, IANS also plans to offer a certification programme for HRA, which may be useful for providers seeking institutional accreditation or remuneration, and is rolling out a programme of approving new HRA courses as they arise. Interested clinicians can seek mentoring from established anoscopists.

Achieving practical training can be challenging given the paucity of trained anoscopists, most of whom are from other specialties. Practice is essential for developing the technical skills required and to begin to recognise lesions. As with all procedures, there is a learning curve, estimated at 120 HRAs in a hands‐on training/proctorship setting [19]. New anoscopists should maintain logbooks and correlate clinical impressions with photos, cytology and histology results. HSIL on cytology but not histology should alert the user that HSIL may have been missed at HRA, as a false‐positive HSIL on cytology is rare [28]. Whilst 50–200 HRAs are generally thought to be required before developing competence, many anoscopists believe that maximal diagnostic acumen occurs at around 500 procedures [20, 29, 30]. HSIL diagnosis rates improve with both anoscopist and pathologist training [29].

Widespread variation exists in the practice of HRA because of the relative ‘newness’ of the procedure, variable practices among specialties that offer it and differences in healthcare systems [20]. A survey of American Colorectal Surgeons published in the sexual health literature in 2014 found that of the 18% of surgeons that replied, one in three reported performing HRA, only 31% used acetic acid and magnification and only 46% had received formal training [31]. In recognition of widespread variability and a lack of trainers, IANS have developed expert consensus guidelines defining minimum competencies and clinical standards [20]. They identify training in HRA to include course attendance, knowledge of anorectal anatomy and HPV‐associated anal disease, observation of HRA, proctorship and mentorship, multidisciplinary discussions and patient feedback. Practical minimum standard quality assurance metrics are described in detail, with a summary in Table 3. New anoscopists can use these metrics as benchmarks and audit their prospectively maintained logbooks to assess performance and modify their practice [20]. Rigorous training was required for anoscopists involved in the ANCHOR study [14], in which the benchmark was to identify HSIL in at least 35% of never‐screened MSMHIV aged 35 or older.

**TABLE 3:** International Anal Neoplasia Society quality assurance metrics for HRA practice.

Metric	Number
Number of HRA's performed per year	50 of which HSIL is detected in 20 cases
Anal cytology in high‐risk patients	Technically unsatisfactory sample on first attempt in < 5% of cases
Histologic HSIL found in cytologic HSIL	≥ 90% of cases
Duration of HRA should be	< 15 min in greater than 90% of cases
Problematic pain or bleeding	≤ 10% of patients

*Note:* Quality assurance metrics published by the International Anal Neoplasia Society [20].

In Australia and Aotearoa New Zealand, formal credentialing pathways for HRA are still evolving. Clinicians typically obtain training through a combination of international courses (including IANS certification), supervised practice with experienced anoscopists and local institutional credentialing processes. Collaboration with established centres, either locally or internationally, remains essential to facilitate mentorship and ensure safe implementation of services.

## 4 Funding and Business Case Considerations

Access to public funding to establish an anal cancer screening service can be difficult, especially given the rarity of anal cancer within the general population. Despite HRA being practised globally for decades, it remains an uncommon procedure in many parts of the world and can evoke scepticism in many units unfamiliar with the practice. In Australia, access to public funding for delivering HRA may also be hindered by a lack of specific item numbers. An application has been submitted to the Medical Services Advisory Committee, which is awaiting a decision [32].

Upfront costs to purchase HRA equipment are another potential barrier. This may be overcome by sharing pre‐existing equipment, such as colposcopes that may be used by Gynaecology and lasers, often used in theatre by Plastics or ENT. Whilst initial expenditure may be greater to establish an HRA service based in the outpatient setting, this is likely to be cheaper in the long term than performing HRA through theatres.

Anal cancer is expensive—the average cost of treating a case from referral through to either completion of follow‐up or death was estimated to be £16 473 in 2014 and will likely increase in view of the recent FDA approval of immunotherapy [33]. Even with the need for multiple HRAs, screening costs are likely to be much lower than treatment costs per case, especially given the potential for early detection and less intensive treatment of early‐stage invasive disease. Further supporting this case is that in MSMHIV, HSIL progressing to cancer is not uncommon, occurring at a rate of 1 in 263–377 per year [34, 35]. The ANCHOR study proved that 57% of these cases can be prevented by treating HSIL [14].

## 5 Discussion and Conclusions

HRA is an effective and well‐tolerated tool in the detection and treatment of anal HSIL, with high‐quality evidence that treating HSIL reduces progression to anal cancer in PLWHIV [14]. Despite these benefits, important limitations warrant consideration. A proportion of HSIL lesions regress spontaneously, raising concerns about overdiagnosis and overtreatment, and treatment may be associated with pain, bleeding and psychological distress. However, a recent systematic review on HSIL treatment modalities found that significant complications such as stenosis and incontinence only occur with surgical excision, which has largely been superseded by ablative techniques, which are overall safe [23]. Furthermore, the number needed to treat to prevent one case of anal cancer remains relatively high even among PLWHIV, and is not well defined in other populations [36]. Uncertainty is further compounded by the lack of clearly established screening intervals and long‐term surveillance endpoints, making a shared decision‐making approach essential where recommendations are consensus, rather than trial‐based.

The ANCHOR study demonstrated a significant reduction in progression to anal cancer in PLWHIV treated for HSIL, supporting the role of HRA in screening, diagnosis and treatment within this group [14]. However, the magnitude of benefit and its generalisability to other populations remain uncertain. While adjunctive tools such as HR‐HPV testing and anal cytology are widely available, access to trained HRA practitioners is limited. Increased awareness among patients and clinicians, combined with the need for ongoing surveillance and repeat procedures, is driving a rapid rise in demand for HRA services.

This demand highlights substantial workforce and training challenges. HRA is a specialised skill requiring significant experience, with estimates suggesting 120–500 procedures are needed to achieve proficiency [19, 20, 29, 30]. Limited access to experienced mentors and proctorship further constrains workforce expansion. Moreover, the relative rarity of anal cancer, even in high‐risk groups, creates tension between maintaining sufficient procedural volume for competence and scaling up service capacity.

Given these constraints, HRA is likely to remain a subspecialised service, delivered through multidisciplinary collaboration involving colorectal surgery, gynaecology and infectious diseases. A centralised model, with expertise concentrated in high‐volume centres supported by clear referral pathways, may represent the most sustainable approach. Such systems should also ensure appropriate support for clinicians serving regional and remote populations.

To support future service provision, exposure to HRA should be incorporated into colorectal training to ensure that all trainees develop a foundational understanding of anal cancer screening and HSIL management. Trainees with a specific interest should be encouraged to pursue advanced training in high‐volume centres. Strong inter‐institutional collaboration will be essential to facilitate training opportunities and workforce development. In the context of growing demand, colorectal surgeons are well‐positioned to contribute to and lead the evolution of anal cancer screening services.

## Author Contributions

**Matthew Joseph Marino:** writing – original draft, conceptualization, writing – review and editing, visualization, data curation. **Benedict Blacket:** writing – review and editing, writing – original draft, conceptualization. **Richard Clive Turner:** conceptualization, writing – review and editing, supervision, writing – original draft. **Tamzin Cuming:** conceptualization, writing – review and editing, supervision, writing – original draft. **David R. C. James:** conceptualization, writing – review and editing, supervision, resources, visualization, writing – original draft.

## Disclosure

The authors have nothing to report.

## Data Availability

Data sharing is not applicable to this article as no datasets were generated or analysed during this study.
